# Transcriptomic Analyses Reveal 2 and 4 Family Members of Cytochromes P450 (CYP) Involved in LPS Inflammatory Response in Pharynx of *Ciona robusta*

**DOI:** 10.3390/ijms222011141

**Published:** 2021-10-15

**Authors:** Aiti Vizzini, Angela Bonura, Laura La Paglia, Antonino Fiannaca, Massimo La Rosa, Alfonso Urso, Manuela Mauro, Mirella Vazzana, Vincenzo Arizza

**Affiliations:** 1 Dipartimento di Scienze e Tecnologie Biologiche, Chimiche e Farmaceutiche-Università di Palermo, Via Archirafi 18, 90128 Palermo, Italy; manuela.mauro01@unipa.it (M.M.); mirella.vazzana@unipa.it (M.V.); vincenzo.arizza@unipa.it (V.A.); 2 Istituto per la Ricerca e l’Innovazione Biomedica-Consiglio Nazionale delle Ricerche, Via Ugo La Malfa 153, 90146 Palermo, Italy; angela.bonura@irib.cnr.it; 3 Istituto di Calcolo e Reti ad Alte Prestazioni-Consiglio Nazionale delle Ricerche, Via Ugo La Malfa 153, 90146 Palermo, Italy; laura.lapaglia@icar.cnr.it (L.L.P.); antonino.fiannaca@icar.cnr.it (A.F.); massimo.larosa@icar.cnr.it (M.L.R.); alfonso.urso@icar.cnr.it (A.U.)

**Keywords:** NGS, cytochrome P450, miRNA, *Ciona robusta*, LPS

## Abstract

Cytochromes P450 (CYP) are enzymes responsible for the biotransformation of most endogenous and exogenous agents. The expression of each CYP is influenced by a unique combination of mechanisms and factors including genetic polymorphisms, induction by xenobiotics, and regulation by cytokines and hormones. In recent years, *Ciona robusta*, one of the closest living relatives of vertebrates, has become a model in various fields of biology, in particular for studying inflammatory response. Using an in vivo LPS exposure strategy, next-generation sequencing (NGS) and qRT-PCR combined with bioinformatics and in silico analyses, compared whole pharynx transcripts from naïve and LPS-exposed *C. robusta*, and we provide the first view of cytochrome genes expression and miRNA regulation in the inflammatory response induced by LPS in a hematopoietic organ. In *C. robusta*, cytochromes belonging to 2B,2C, 2J, 2U, 4B and 4F subfamilies were deregulated and miRNA network interactions suggest that different conserved and species-specific miRNAs are involved in post-transcriptional regulation of cytochrome genes and that there could be an interplay between specific miRNAs regulating both inflammation and cytochrome molecules in the inflammatory response in *C. robusta*.

## 1. Introduction

Cytochrome P450 enzymes (CYPs) consist of a large group of haemoproteins that catalyse a wide range of reactions involved in the oxidative metabolism of xenobiotics, such as drugs, pesticides, and environmental chemicals, or in the metabolism of endogenous compounds, such as steroid hormones, fatty acids, eicosanoids, and vitamins [1,2,3,4]. *CYP* genes are widely distributed in all domains of life, from bacteria, archaea, and viruses to higher plants and animals and may be classified in class, group, clan, family and subfamily [5]. The *CYPs* constitute one of the most diverse eukaryotic genes, with a wide complexity within and between species. CYP enzymes use molecular oxygen to modify substrate structure, critical in a huge number of physiological, ecological and toxicological processes. In chordates *CYP* genes, created by the 2R whole genome duplications, 11 distinct clans have been identified, each containing one or more *CYP* gene families. *CYP* constitutes a large gene class descended from a single common ancestor, termed the “cytochrome P450 genesis locus”. Duplicated in tandem of progenitor *CYP* gene gave rise to a set of genes that were precursors of the 11 animal *CYP* clans: *CYP* Clans 2, 3, 4, 7, 19, 20, 26, 46, 51, 74 and mitochondrial [6]. The concept of whole-genome duplication (WGD) during animal evolution is essential background for considering *CYP* gene evolution [7]. Analysis of the *Ciona* genome confirmed the 2R hypothesis [8], as did the recent sequencing of the amphioxus (lancelet) genome [8]. Evolutionarily, the 2R events have been bracketed in time between the divergence of tunicates from vertebrates and the origin of the gnathostomes (jawed vertebrates) [9,10], and in addition, a third-round (3R) has occurred in the vertebrate line leading to ray-finned (actinopterygian) fishes including zebrafish and medaka [11,12]. Not all animals have the 11 animal *CYP* clans, in fact, the Ecdysozoa (insects, crustaceans, nematodes) only have Clans 2, 3, 4 and mitochondrial and other protostomes such as molluscs and annelids may have additional clans (7, 20, 26 and 51) [13].

In humans, 57 functional genes and 58 pseudogenes have been identified [14], grouped according to their sequence similarity into 18 families and 44 subfamilies. Expression of *CYPs* is influenced by genetic polymorphisms, induction by xenobiotics, regulation by cytokines, hormones, epigenetic factors, including DNA methylation, histone modification, and ncRNA regulation [15,16]. Furthermore, it was demonstrated that LPS regulated the xenobiotic-induced expression of representative *CYP* genes and increased the levels of mRNAs of Tumour necrosis factor (TNF) and interleukin (IL)-1. The changes in these cytokines, which are well-known pro-inflammatory mediators, correlate with the changes in *CYP* expression and enzymatic activity during infection and inflammation [17,18,19,20].

The ascidian *Ciona robusta*, which shows a close phylogenetic relationship with vertebrates [21,22,23,24], is a powerful animal model to study comparative immunity [25] and the evolution of transcriptional and post-transcriptional regulatory mechanisms in genes expression. In *C. robusta* a strong inflammatory response can be induced by inoculating LPS into the body wall (tunic and contiguous pharynx). The pharynx (haematopoietic organ) occupies a large part of the adult body and consists of two epithelial monolayers perforated by dorso-ventrally aligned rows of elongated elliptical, ciliated stigmata [26,27] enclosed in a mesh of vessels (also called transversal and longitudinal bars), where the haemolymph, containing abundant mature and immature haemocytes, flows. Recently, *C. robusta* has become an excellent model for the study of distinct functional categories of immune genes such as effector genes as mannose-binding lectin-like (*Mbl-like*) [28], galectin-like (*Gal-like*) [29] and cytokines as interleukins 17 (*Il-17*) [30], Tumour growth factor (*Tgf-ß*) [31] and Macrophage migration inhibitory factors (*Mif1* and *Mif2*) [32] that are coordinately and temporally regulated by mechanisms that control transcriptional and post-transcriptional regulation. In the *C. robusta* pharynx, the Tgf-β, Wnt, Hedgehog and FoxO pathways, which are involved in tissue homeostasis, resulted in regulated in an integrated network by different conserved and species-specific miRNAs, pseudogenes and 3′UTR mRNA elements [33]. 

In the present study, we used an in vivo strategy to compare *C. robusta* pharynx transcripts from naïve and LPS-exposed and qRT-PCR, and we indicate the involvement of 2 and 4 family members of Cytochrome in inflammation. Moreover, miRNA targets prediction analyses of cytochrome genes and immune genes suggest that, as humans, conserved and species-specific miRNAs can be involved in post-transcriptional control in the LPS-mediated inflammatory response.

## 2. Results

### 2.1. Next-Generation Sequencing Transcriptomics Analysis of Ciona robusta Highlights the Effects of LPS on Cytochrome P450 Transcripts Involved in Inflammation

**Figure 1:**
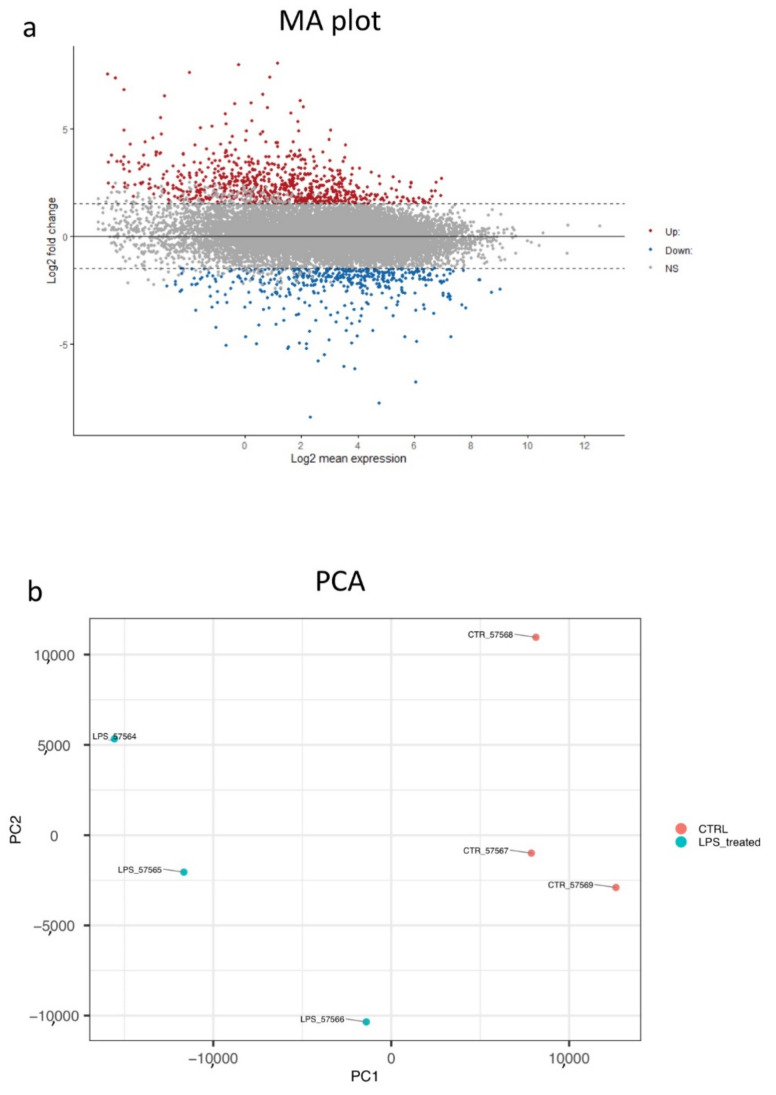
MA plot (**a**). X axis reports the Log2 mean expression of genes; Y axis reports the log2FC (threshold = 1.5). Red points are upregulated genes, blue points are down-regulated genes. PCA plot (**b**). Principal Component 1 (PC1) and PC2 of normalized data are reported in Y and X axis. Blue points represent *C. robusta* exposed to LPS (*n* = 3) and red points represent untreated ascidians (*n* = 3). The number of total points in PCA is the number of replicates used for NGS experiment (*n* = 6).

Next-generation sequencing (NGS) analysis of the differential gene expression in immunocompetent organ (pharynx) under physiological and challenging conditions (4-h LPS exposure) treatment in vivo were performed to profile the *C. robusta* inflammatory response. NGS identified 16,504 transcripts annotated in the Ensembl database (ensemble.org), and for each of them, log-fold changes, *p*-values and adjusted *p*-values were calculated (data not shown). The analysis evidenced 1227 differentially expressed (DE) genes (up-regulated and down-regulated) (Figure 1) [34]. They were filtered for the Log2FoldChange (LogFC) threshold of 1.5, with a *p*-value and adjusted *p*-value thresholds <0.05.

**Figure 2:**
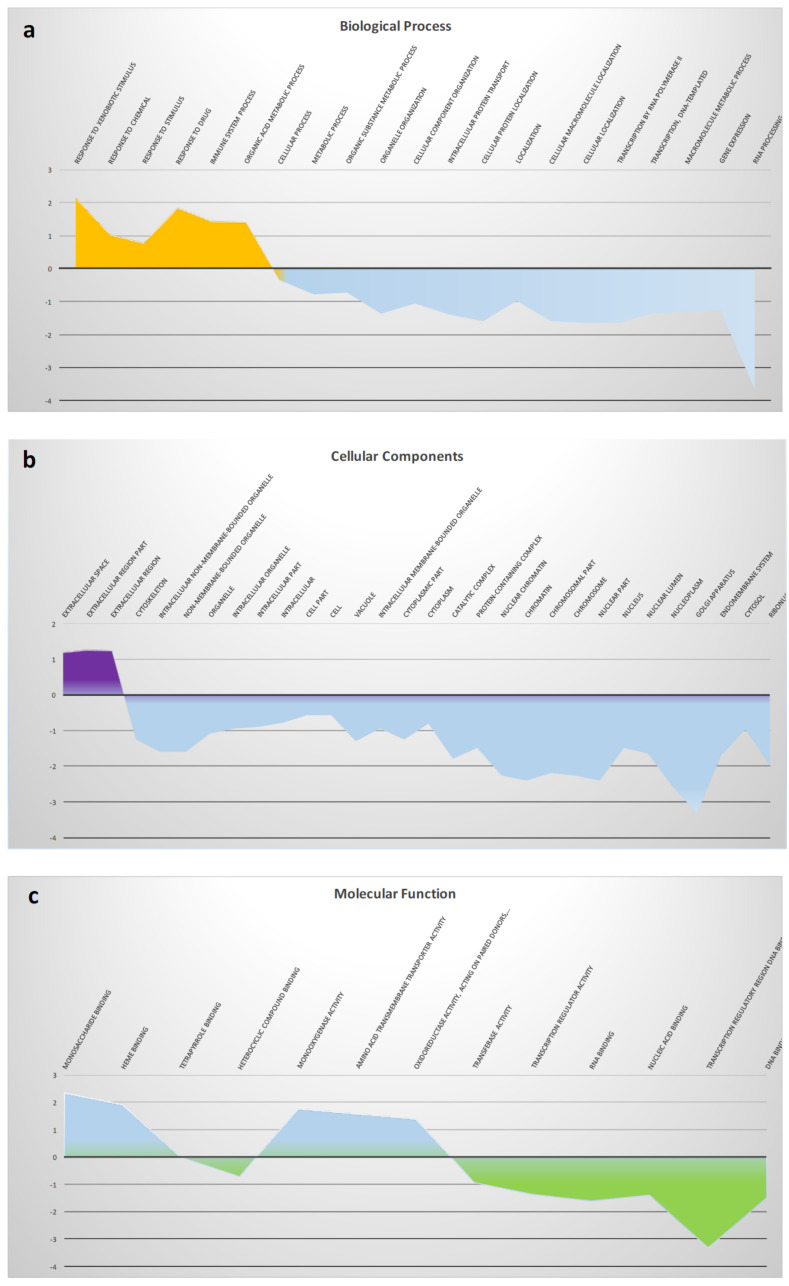
GO analysis of the three different GO subclasses for all the deregulated genes of NGS. (**a**) Biological Process (BP), Cellular Components (CC) (**b**) and (**c**) Molecular Functions (MF) are showed. Y axis: log10 fold enrichment; X axis: respectively BP, MF and CC ontology classes.

For functional annotations, unigenes (1227) were aligned to Gene Ontology (GO) terms based on the Protein ANalysis THrough Evolutionary Relationships (PANTHER) classification system for gene ontology annotations (pantherdb.org, 16.0 release); that it is connected to the Clusters of Orthologous Genes (COG) database (geneontology.org, release February 2021). The *C. robusta* transcripts were classified in the three GO subcategories: (i) GO Biological Processes (BP) (Figure 2a); (ii) GO Molecular Functions (MF) (Figure 2b); (iii) GO Cellular Components (CC) (Figure 2c).

Between genes responsive to LPS, different positively (+) enriched classes belonging to the following GO terms were evidenced: (i) Biological Processes: response to xenobiotic stimulus, response to chemicals, response to stimulus, response to drugs, response to immune system processes and organic acid metabolic processes; (ii) Cellular Components: extracellular space, extracellular region and extracellular region part; (iii) Molecular Function: monosaccharide binding, heme binding, tetrapyrrole binding, monooxygenase activity, amino acid transmembrane transport, oxidoreductase activity.

These results strongly indicated the presence of DE genes linked to Cytochrome P450 (*Cyp 450*) families and inflammatory response. 

Indeed cytochrome P450 enzymes catalyze a wide range of reactions involved in the oxidative metabolism of xenobiotic, or in the metabolism of endogenous compounds; moreover, they use molecular oxygen to modify substrate structure, critical in a huge number of physiological, and toxicological processes. 

Finally, the expression of xenobiotic-induced cytochromes is mediated by LPS stimulation, which also increases different cytokines which are well-known pro-inflammatory mediators, thus correlating a potential link between these molecules and immunity response processes.

A deeper investigation on DE genes evidenced different families of cytochromes that resulted in deregulation in NGS experiments. In particular, *cytochrome P450* components of 2 and 4 families were deregulated (Table 1). 

### 2.2. Alignment and Phylogenetic Analysis of C. robusta Cytochrome P450 LPS Deregulated Genes

**Figure 3:**
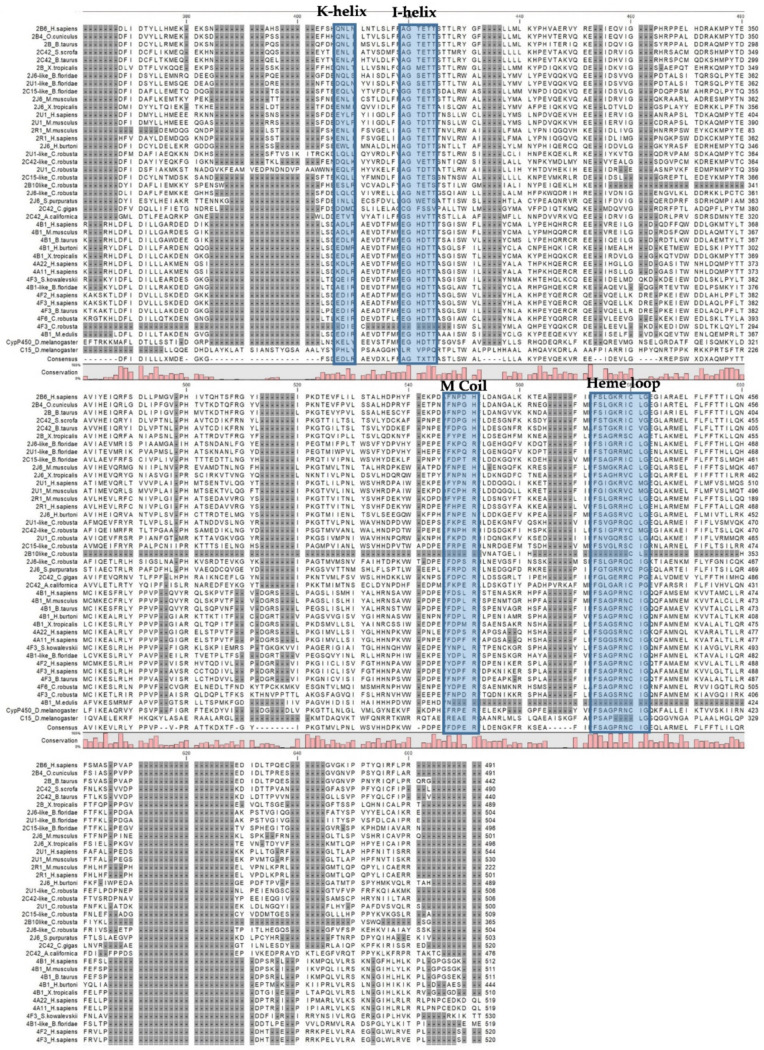
Multiple amino acid sequence alignment of Cytochrome P450 2 and 4 family members from invertebrates, vertebrates and *C. robusta*.

The Cytochrome P450 LPS deregulated genes (Table 1) were aligned with vertebrate and invertebrate *Cytochrome P450* 2 and 4 family members (Figure 3). Multiple alignments showed that all the 2 and 4 family members of *C. robusta* Cytochrome P450 possess the characteristic conserved motif of Cytochrome P450 (Figure 3; Table 2). The cysteine residue coordinating heme iron result conserved in all the sequences, in a consensus sequence well-conserved in cytochrome P450 (FXXGX(H/R)XCXG), together with the EX1X2R motif located in helix K involved in salt bridge interactions, that are important for its tertiary structure and the correct incorporation of the heme cofactor, (AG)Gx(DE)T(TS) located in I helix and FDDER located in M Coil (Figure 3; Table 2).

**Figure 4:**
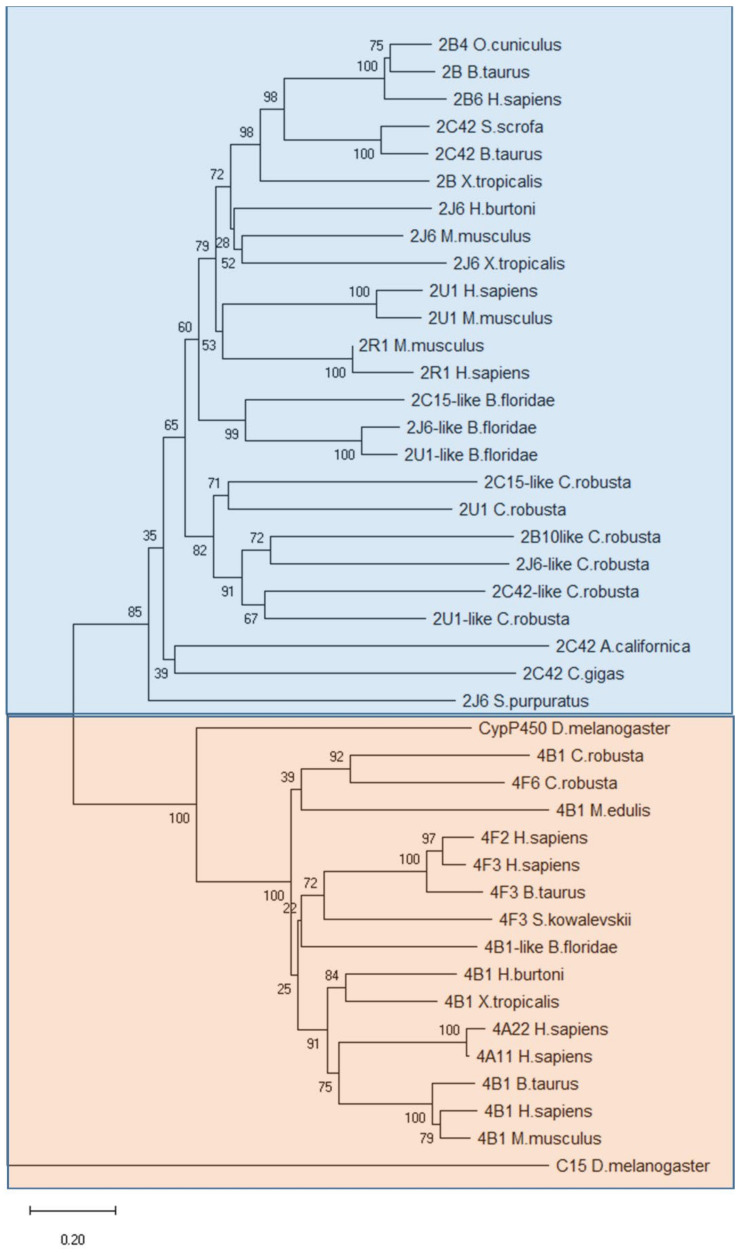
Phylogenetic tree of Cytochrome P450 2 and 4 family members in vertebrates and invertebrates. The tree was constructed by the neighbour-joining method and bootstrap analysis. The bootstrap value indicates the number of particular node occurrences per 1000 trees, as generated by bootstrapping the sequences, expressed as a percentage. The bar indicates the number of amino acid residue substitutions per site.

To understand the evolutionary relationship between the known 2 and 4 family members of Cytochrome P450 in vertebrates, invertebrates and *C. robusta*, we performed a phylogenetic analysis looking for orthologous sequences retrieved in the NCBI database (https://www.ncbi.nlm.nih.gov) (Accessed on 15 June 2021). Orthologous molecules were aligned using the ClustalW algorithm and neighbour-joining trees were constructed. A phylogenetic tree was constructed using the amino-acid sequence of *D. melanogaster* as an out-group (MEGA X program) (Figure 4). The Cytochrome P450 in vertebrates and *C. robusta* formed two distinct major clusters. The first cluster includes the group of vertebrates, invertebrates and *C. robusta* Cytochrome P450 belong to 2 family members, the second group includes the group of vertebrates, invertebrates and *C. robusta* cytochrome P450 belong to 4 family members (Figure 4). The phylogenetic analysis supported the idea of a conserved evolution of Cytochrome P450 from a common ancestral gene among protochordates and vertebrates.

### 2.3. Differential Gene Expression of Cytochrome P450 Genes in Different Tissues

**Figure 5:**
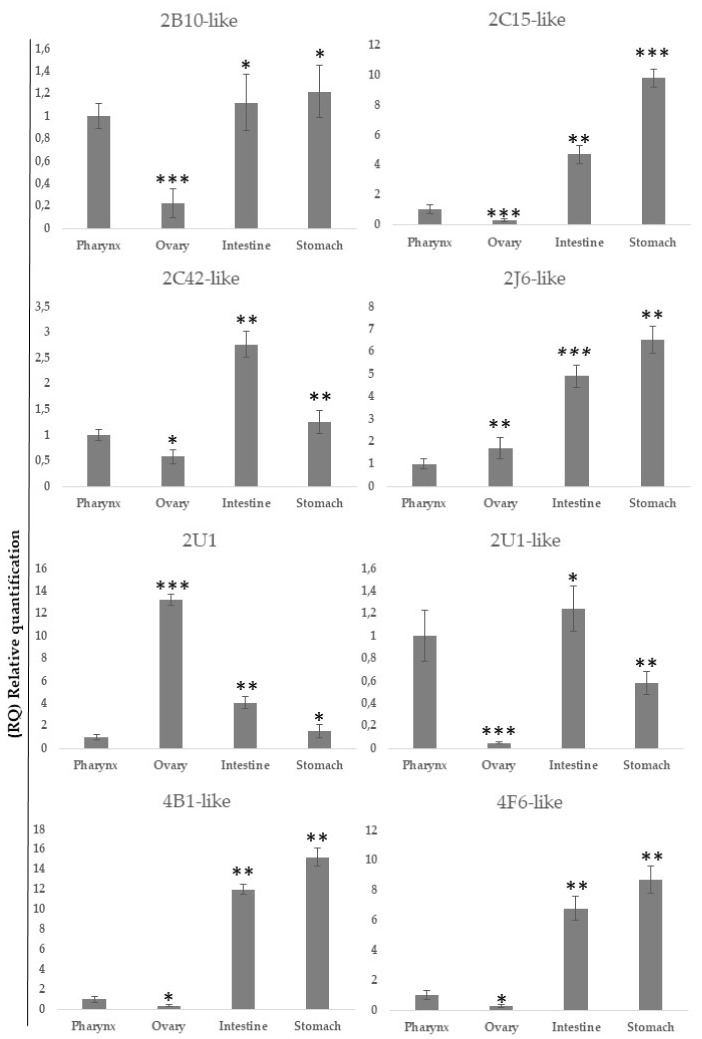
qRT-PCR analysis: Tissue expression of Cytochrome *p* 450 of *C. robusta*. Values, plotted as mean ± SD, were inferred from four ascidians. * *p* < 0.05, ** *p* < 0.005, *** *p* < 0.001.

The spatial expression pattern of Cytochrome mRNA (Table 1) in adult *C. robusta* was investigated by qRT-PCR analysis of total RNA samples from different tissue involved in inflammatory response (pharynx, ovary, stomach and intestine). As shown in Figure 5, Cytochrome P450 expression was higher in the stomach and the intestine, and lower in the ovary and pharynx tissue; 2B10, 2C15, 2J6, 4B1 and 4F6 family members expression was higher in the intestine and the stomach, and lower in the ovary and pharynx tissue; 2U1 family member expression was higher in the ovary, and lower in the intestine, the stomach and pharynx tissue; 2U1-like family member expression was higher in the pharynx and intestine and lower in the stomach and ovary tissue.

### 2.4. Analyses of the Expression of Cytochrome 450 and Cytokines Genes under LPS Exposure

**Figure 6:**
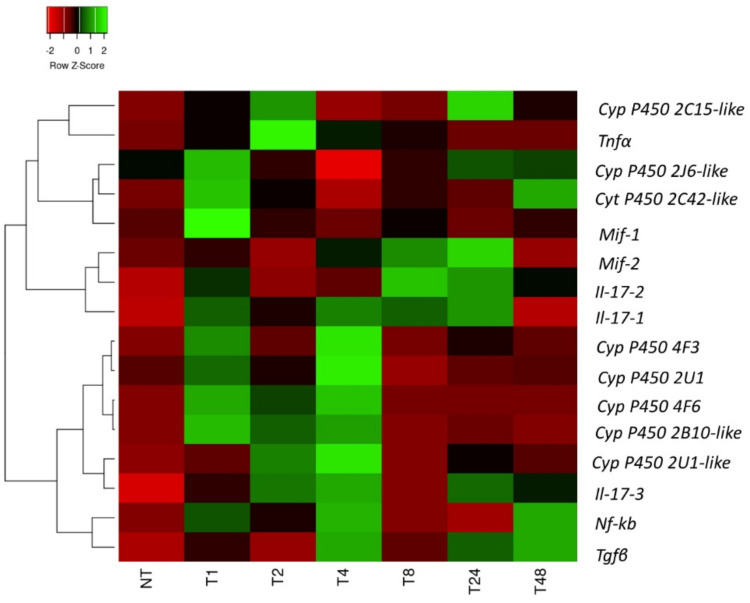
Heatmap based on the qRT-PCR analysis of the differentially expressed *Cytochromes P450*, *Nf-κB* and *cytokines* at different times of exposure to LPS (1–48 h). Time course of gene expression in the pharynx of *C. robusta* exposed to LPS compared with the gene expression in untreated ascidians. To compute the heatmap was chosen to use the Complete linkage as clustering algorithm, and the Pearson correlation as distance measurement method. Values are represented according to the z-score, which is measured in terms of standard deviations from the mean.

Analyses of the time-course expression of Cytochrome and Cytokines in the pharynx inflammatory response induced by LPS in *C. robusta* were performed at time points from 0 to 48 h post-LPS challenge by qRT-PCR (Figure 6). The heatmap shows that cytochrome transcripts were significantly modulated in response to LPS during the 48-h period of LPS exposure (*p*-value < 0.05). Based on the expression patterns of the transcripts, two major clusters were highlighted: the first includes pro-inflammatory cytokines *Mif1*, *Mif2, Il-17–1*, *Il-17–2*, and *Tnf-α* and *Cyp450 2C15, 2J6, 2C42* and the second comprises *Nf-κB* and *Tgf-β, Il-17–3* and Cyp450 2U1, 2U1-like, 2B10-like, 4B1, 4F6. Specifically, the heatmap highlighted that the inflammatory cytokines *Mif1*, *Il-17–1*, *Il-17–3*, and *Tnf-α* were upregulated between 1 and 4 h of LPS exposure (*p*-value < 0.05) and *Tnf-α* reached its maximum expression level after 2 h of LPS exposure. Notably, *Nf-κB* and transforming growth factor β (*Tgf-β*) transcripts displayed a significant increase after 4 h of LPS exposure (*p*-value < 0.05). On the other hand, after 8 h of exposure, *Il-17–1*, *Il-17–2* levels began to increase at 1 h. *Nf-κB* and *Tgf-β* show a second significant increase after 48 h of exposure (*p*-value < 0.05). *Cyp450 2C15, 2J6, 2C42* mRNA were upregulated between 1 and 2 h of LPS exposure, while *Cyp450 2U1,2U1-like, 2B10-like, 4B1, 4F6* were upregulated between 1 and 4 h of LPS exposure. These findings suggest an involvement of cytokines in modulating the expression of Cytochromes in response to LPS exposure.

### 2.5. miRNA-Target Interaction Prediction

miRNA-target interaction of deregulated genes belonging to cytochrome (Table 1) and inflammation was investigated, by using the miRNA Target Interactor Predictor (miRNATIP) algorithm to explore how deregulation of Cytochrome P450 enzymes may be driven by non-coding RNA intervention during the inflammation process. miRNA-target prediction evidenced several miRNAs interacting with both DE genes linked to the Cytochrome P450 family and inflammation. A total of 10,002 interactions were predicted for DE Cytochromes and 3905 interactions were predicted for DE cytokines. Results were then filtered for the lowest free energy values (free energy < −12 Kcal/mol). Table 3 shows a detailed list of miRNA-target interactions, showing just interactions with more than two mRNA targets for each miRNA are showed. A total of 42 of all miRNAs identified by the miRNATIP algorithm were common to the two differentially expressed genes classes, and they belong to 36 different miRNA families. Seven of 36 families were conserved through species and 29 were species-specific. 

The distinction between conserved and specie-specific miRNAs was made using different web services (MirGeneDB 2.0 (https://mirgenedb.org, MirGene DB 2.0 release) (Accessed on 21 July 2021); miREval 2.0 (http://mimirna.centenary.org.au/mireval/) (Accessed on 21 July 2021)and miRBase database (http://www.mirbase.org, release October 2018) (Accessed on 21 July2021) that study evolution pattern of conserved *C. robusta* homologues miRNA in the animal genome. 

See Table 4 for a detailed list of predicted miRNAs, conserved through species and species-specific, that are common to the Cytochrome P450 and inflammation genes. See supplementary material for all miRNA-target interactions (Supplementary Materials).

### 2.6. Cytochrome and Inflammation miRNA-Target Network Reconstruction

**Figure 7:**
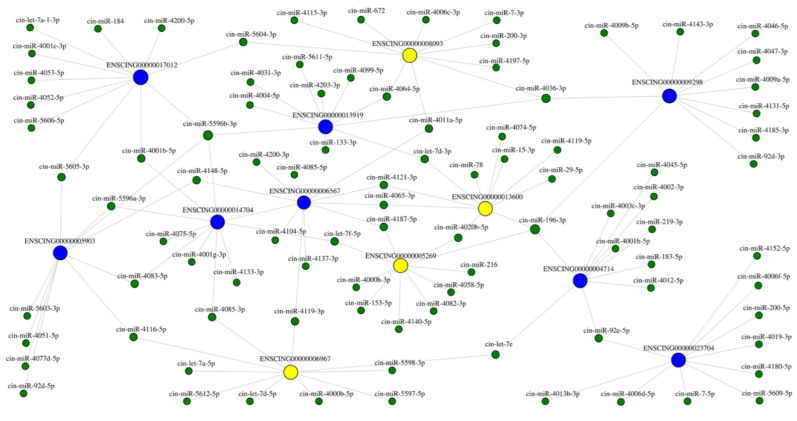
Interaction network between miRNAs (green), DE Cytochrome genes (blue) and DE inflammatory cytokines (yellow). The network analysis was performed using R package and R studio, and miRNA prediction results were integrated into the two different networks of DE Cytochrome genes and inflammatory cytokines.

miRNA-target interaction network reconstruction allows to easily visualise relevant interactions between different molecules. To build the graph, different steps were required: first, DE genes of Cytochromes and inflammation networks were downloaded into the Search Tool for Recurring Instances of Neighbouring Genes (STRING) database (string-db.org, release 11.5, accessed on 12 August 2021), second, the two different networks produced (cytochromes and inflammation) were imported into Cytoscape app (httos://cytoscape.org, release March 2017) (Accessed on 13 August 2021), to generate graph.ml files, and third, the networks were analysed using R package and R studio (https://www.r-project.org/, release October 2020) (Accessed on 17 August 2021); (https://rstudio. com/products/rstudio/, release October 2020) (Accessed on 17 August 2021), and finally miRNA prediction results were integrated into two different networks (Figure 7).

The miRNA-target network reconstruction put in evidence that some miRNAs interacting with more of two different targets, some regulating different Cytochrome genes, others regulating both genes of Cytochrome and cytokines (Table 3).

Indeed, between conserved miRNAs, cin-let-7d-3p, interacts with ENSCING00000013600 (transforming growth factor beta superfamily signalling ligand (*Tgf-ß-na2*) and ENSCING00000013919 (*Cyp450 2C42-like*). Other members of the same let-7 family also interact with genes of cytochrome and inflammation processes: cin-let-7e interacts with ENSCING00000006967 (*Il-17-1*) and ENSCING00000004714 (*Cyp450 4B1-like*); cin-let-7f-5p interacts with ENSCING00000005269 (*Il-17-3*) and

ENSCING00000004714 (*Cyp450 4B1-like*); cin-miR-92e-5p interacts with 

ENSCING00000004714 (Cyp450 4B1-like) and ENSCING00000023704 (Cyp450 2B-10, male-specific-like).

Interestingly, cin-miR-196-3p interacts with four different targets: 

ENSCING00000013600 (*Tgf-ß-na2*), ENSCING00000005269 (*Il-17-3*), ENSCING00000004714 (*Cyp450 4B1-like*) and ENSCING00000009298 (*Cyp450 2U1-like*).

Three species-specific miRNAs (cin-miR-5596b-3p, cin-miR-4085-3p and cin-miR4036-3p) also have more common targets. The first interacts with ENSCING00000004714 (*Cyp450 4B1-like*), ENSCING00000013919 (*Cyp450 2C42-like*), ENSCING00000017012 (*Cyp450 2J6-like*) and ENSCING00000005903 (*Cyp450 2U1*); the second interacts with ENSCING00000022988 *(Cyp450 4F4-like),* ENSCING00000004714 (*Cyp450 4B1-like*) and ENSCING00000006967 (*Il-17-1*); and the third interacts with ENSCING00000009298 (*Cyp450 2U1-like*), ENSCING00000013919 (*Cyp450 2C42-like*) and ENSCING00000008093 (transforming growth factor beta superfamily signalling ligand (*Tgf-ß*-*na1*)). 

These in silico pieces of evidence let us hypothesize that there could be an interplay between specific miRNAs regulating both the inflammation process and cytochrome molecules. 

### 2.7. Transcription Factor Orthologue Identification of Cyp450 Response Elements

After network reconstruction, Cyp450 gene transcriptional regulation by specific transcription factors (TFs) was investigated. Indeed, as known by scientific literature, TFs bind gene response elements and activate mRNA transcription of target genes. 

To this aim we searched for orthologue genes in *C. robusta* through the National Center of Biotechnology Information (NCBI) (https://www.ncbi.nlm.nih.gov/gene, 244 release, accessed on 15 June 2021), orthologue database (orthoDB) (orthoDB; https://www.orthodb.org/v9, release v10.1) (Accessed on 20 August 2021) and through REGULATOR tool (https://www.bioinformatics.org/regulator, release 27.0) (Accessed on 20 August 2021), hypothesising that different TFs can potentially interact with *Cyp450* response elements, to regulate mRNA Cyp450 expression.

**Figure 8:**
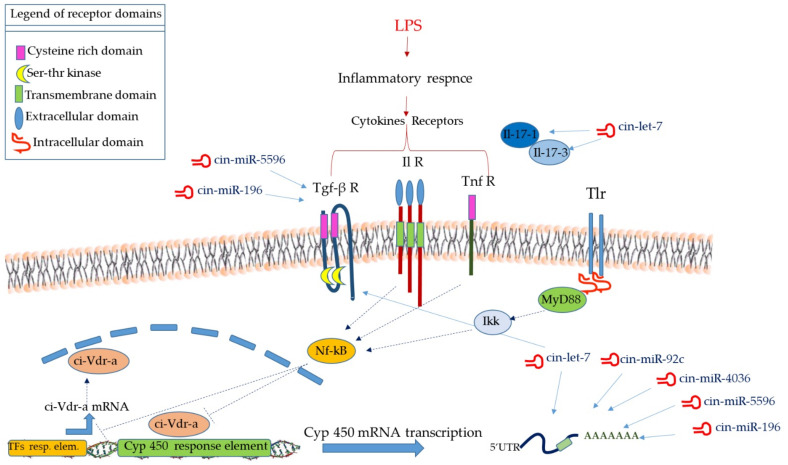
Schematic representation of potential transcriptional and post-transcriptional mechanisms that could regulate Cytochrome P450 enzymes in inflammation: conserved and species-specific miRNAs regulate the transcription of different Cytochrome genes, and also the signalling pathway linked to inflammatory-like reactions and cytokines. Moreover, TFs, as ci-Vdr-a can potentially bind Cyp450 response elements, activating Cyp450 transcription.

Transcription factors identified in *C. robusta* genome are ci-Hnf-1 (ncbi gene ID: 778644), ci-Hnf-4 (ncbi gene ID: 778645), ci-AhR (ncbi gene ID: 778536), ci-Rxr (ncbi gene ID: 778746), ci-C/Ebp (ncbi gene ID: 778557) and ci-Vdr-a (ncbi gene ID: 778791). In silico analyses of TF binding sites prediction were finally carried out. Site Tracking and Recognition (SiTaR) tool (https://sbi.hki-jena.de/sitar/, V 0.1) (Accessed on 24 August 2021) was applied to all mentioned TFs and deregulated Cytochrome genes produced by NGS. Result analysis showed that just the ci-Vdr-a transcription factor potentially interacts with most deregulated cytochrome genes analysed (see Supplementary Material for detailed analysis results). Figure 8 shows a schematic representation of the potential transcriptional regulation of *Cyp450* genes in response to TFs and non-coding RNAs.

## 3. Discussion

Cytochrome P450 (CYP) enzymes are an ancient superfamily of monooxygenase proteins found in all domains of life [35]. Genome sequencing of diverse species showed ~12,000 known *CYP* genes [36]. Cytochromes are expressed in many different tissues of the human body. They are found mostly in intestinal and hepatic tissues. In this study by an in vivo LPS exposure strategy and next-generation sequencing (NGS), we found that in *Ciona robusta*, the closest phylogenetically to vertebrates, 57 gene encoding for Cytochrome belong to 2,3,4,10,26 family members and 2B10, 2J6, 2C15, 2C42, 2U1, 2U1-like and 4 B1,4F6 subfamily members result involved in inflammatory response LPS induced. The *CYP2* family has been primarily studied in vertebrate species and results in the largest and most diverse of the vertebrate *CYPs* [14,37]. Comparison of *CYP2* subfamilies in vertebrate genomes suggests there are lineage-specific *CYP2* subfamilies in mammals (*CYP 2A, 2B, 2C, 2E, 2F, 2G, 2S, 2W*), fish (*CYP 2K, 2M, 2N, 2P, 2V, 2X, 2Y, 2Z, 2AA, 2AD, 2AE*), birds (*CYP2H*), and amphibians. The *CYP2U* and *CYP2R* genes were present in the vertebrate ancestor and are shared across all vertebrate classes; *CYP2D* and *CYP2J* are not found in actinopterygian (ray-finned fish) species [37]. Members of this family were found in arthropods and crustacean species, such as *CYP2L* in lobster [38], and a significant number of *CYP2*-*like* genes were found in the sea urchin [39]. In *C. robusta* LPS Analyses of the time-course expression of Cytochrome P 450 and Cytokines in the pharynx inflammatory response induced by LPS showed that *Cyp450 2C15, 2J6, 2C42* were upregulated between 1 and 2 h. In contrast *Cyp450 2U1,2U1-like, 2B10-like, 4F3,4F6* were upregulated between 1 and 4 h. While pro-inflammatory cytokine *Tnf-α* reached its maximum expression level after 2 h of LPS exposure and *Nf-κB* and transforming growth factor β (*Tgf-β*) transcripts displayed a significant increase after 4 h of LPS exposure. These findings suggest an involvement of cytokines and *Nf-κB* in downregulation of the Cytochromes response upon LPS exposure after 4h. In humans, rats, and mice [40,41,42] variations in hepatic *CYP* expression are common responses to infection and inflammation. Morgan [43] reported that levels of constitutively expressed *CYP* genes were downregulated in the rat liver when inflammation was induced with lipopolysaccharide (LPS). Furthermore, the changes of pro-inflammatory mediators TNF and IL-1 correlate with the changes in *CYP* expression and enzymatic activity during infection and inflammation [18,19,20]. Increasing these cytokines appears to strongly affect the pathways regulating inducible *CYP* expression and LPS markedly alters *CYPs’* expression levels during inflammation as *Cyp2c29 and Cyp 2B10 mRNA* in the liver [44]. Lipopolysaccharide (LPS) treatment of rats suppresses *Cyp 4F4* and *4F5* expression by 50 and 40%, respectively. In this animal model, a change in the expression of *Cyp 4F4* and *4F5* mRNA was observed at 24 h compared to the controls. At time points after 24 h the expression of both isoforms increases dramatically reaching the highest levels at 2 weeks post-injury. These results are consistent with the notion that immediately after injury, concentrations of leukotriene and prostaglandin mediators immediately after injury are elevated by decreased *Cyp 4F* concentrations. Increased *Cyp 4F* expression leads to diminished concentrations of leukotriene and prostaglandin mediators and then to recovery and repair [45] In vitro models NF-kB can control the expression of *CYP1A1, CYP2B1, CYP2C11, CYP2D5, CYP2E1*, and *CYP3A7* via interaction with the promoters of these genes, leading to downregulation [46]. In vitro models show that distinct mechanisms are involved in the downregulation of CYP enzyme expression and Cytokine-mediated alteration of gene transcription. De jong [47] suggests that they can be the main regulatory mechanism accountable for changing *Cyp450* activity upon inflammation [47]. The mechanisms of downregulation of Cytochromes P450 upon inflammation is an area of intense study. Increasing attention is being given to the potential post-transcriptional mechanisms that could regulate P450 enzymes in inflammation, as the involvement of MicroRNAs. MicroRNAs (miRNAs) inhibit gene expression largely in a posttranscriptional manner by interacting with miRNA response elements (MREs) in the mRNA 3′-UTRs of the target genes [48]. In *C. robusta* the NGS strategy identified transcripts that were classified as non-coding RNA (ncRNA). Of these 201, 90 were miRNAs. Both conserved (cin-let- 7d-5p, cin-mir-153-5p, and cin-mir-92c-5p) and species-specific miRNAs were found involved in post-transcriptional regulation of four pathways (Tgf-β,Wnt, FoxO, and Hh) linked to pharynx tissue homeostasis [33]. Bioinformatics and miRNA-target prediction analysis show that conserved and species-specific miRNAs can be involved in regulating post-transcriptional mechanisms of both gene categories: Cytochrome P450 and cytokines. Particularly cin-let-7 and cin-miR-92 seem vastly involved in post-transcriptional regulation during LPS induced inflammatory processes. In vertebrates, regulation of hepatic CYP levels is involved a wide variety of ligand-activated transcription factors and nuclear receptors. In inflammation, in the regulation of CYP mRNA levels, have involved Transcription factors including the nuclear receptors pregnane X receptor (PXR), the constitutive androstane receptor (CAR), their dimerization partner retinoid X receptor (RXR), the aryl hydrocarbon receptor (AhR) and human nuclear factors (HNFs) that are also responsible for the downregulation of Cytochromes P450 in inflammation. Nuclear Receptors as CAR increase transcription of the human *CYP3A4/5, CYP2C9, CYP2C19*, and *CYP1A2* genes upon drug treatment [49,50,51]. Nuclear receptor PXR and CAR are involved in the mechanism of changes in gene transcription of major Cytochromes in inflammation. Vast evidence shows that inflammation represses PXR levels, leading to downregulation of important CYP enzymes [52,53] and LPS treatment in mice led to functional repression of PXR’s dimerization partner RXR [54]. A likely scenario is that the suppressive action of inflammation on PXR expression is mediated through NF-kB activation since Zhou X et al. showed that NF-kB directly interacts with a functional binding site in the PXR promotor to suppress its transcriptional expression [55]. *C. robusta* genome reveals only a single putative orthologue to vertebrate nuclear receptors PXR which was most similar to mammalian PXRs [56] with the ability to adapt to different ligands [57]. Furthermore, in silico analysis showed that the nuclear transcription factor ci-Vdr-a belongs to a nuclear hormone receptor family that is homologue to human PXR (ncbi ID: 8856), can potentially interact with many of deregulated Cytochrome genes identified by NGS. Thus, we suggest that ci-Vdr-a could potentially act as its orthologue protein PXR, intervening in the transcriptional regulation of Cytochrome genes during the inflammation process.

Concluding, we assume that in *C. robusta* (Figure 8) the modulation of cytochromes belonging to 2B, 2C, 2J, 2U, 4B and 4F subfamilies genes expression during inflammation may proceed through transcriptional downregulation of transcription factors, interference with dimerization/translocation of (nuclear) transcription factors, involvement of NF-kB, and inhibition gene expression in a posttranscriptional manner by interacting with miRNA. The control of Cytochrome P450 gene expression happens in a coordinated manner with the control of gene expression of immune genes as Cytokines and NF-kB according to highly conserved mechanisms during evolution.

## 4. Materials and Methods

### 4.1. Tunicates and LPS Injection

The animal model *Ciona robusta* was formerly classified as *Ciona intestinalis*. Molecular studies have confirmed that *C. intestinalis* constitutes a compilation of species rather than a single speciements [57,58,59,60,61]. *C. robusta* were collected from Sciacca harbour (Sicily, Italy) and were acclimatized and maintained as reported in Arizza et al. [34]. An LPS solution (*Escherichia coli* 055:B5, LPS, SIGMA-ALDRICH, Saint-Louis, MI, USA) was prepared in a sterile salt medium (12 mM CaCl2, 11 mM KCl, 26 mM MgCl2, 43 mM Tris HCl, 0.4 M NaCl, pH 8.0). One hundred microliters of the LPS-containing suspension was injected into the tunic matrix surrounding the pharynx wall (median body region) at a final LPS concentration of 100 μg. *C. robusta* not exposed to LPS (*naïve*) were used as controls. Fragments of pharynx tissue (200 mg) explanted at various times (from 1 to 48 h) and pharynx, ovary, intestine and stomach tissues of *naïve* were immediately soaked in RNAlater tissue collection solution (AMBION, Austin, TX, USA) and stored at −80 °C. Total RNA extraction was performed using an RNAqueous-Midi kit purification system (AMBION, Austin, TX, USA) as reported in Arizza et al. [34].

### 4.2. RNA Sequencing (RNA-Seq)

The RNA purity and quality of total RNA extracted from the pharynx of *C. robusta* that were naïve 3 replicates (*n* = 3) and that were exposed to LPS for 4 h 3 replicates (*n* = 3) were examined by NanoDrop and Agilent RNA 6000 Nano kits on an Agilent 2100 Bioanalyser (AGILENT, Santa Clara, CA, USA), respectively. High-quality RNA samples (A260/A280 = 1.9–2.1, RIN _ 7) were used for cDNA library construction. RNA sequencing (RNA-Seq) was performed by BMR Genomics (Padua, Italy) on an Illumina platform in a single-end format 75 bp (1_75 bp) containing ~40 million _ 10% of reads/sample [34]. All transcripts produced by NGS were annotated by Ensembl database (https://www.ensembl.org/index.html, release August 2020) (Accessed on 3 March 2021). Differential expression between treated (4hrs LPS induction) and untreated genes was performed by BMR genomics [34]. It was performed using edgeR software. It allows to estimate the negative binomial variance parameter globally across all genes. All the data were then normalized by setting the false discovery rate (FDR) to ≤0.05 and the absolute value of the log2 fold change (logFC) to 1.5. A MA plot was performed to visualize the up-regulated and down-regulated genes from RNA-sequencing. The plot visualizes the differences between measurements taken in two samples, by transforming the data onto M (log ratio) and A (mean average) scales, then plotting these values. Results were calculated using ggpubr pakage of CRAN library (https://cran.r-project.org/web/packages/ggpubr/index.html) (Accessed on 30 September 2021).

To visualize the data, the log2 mean expression is calculated, which is the mean of values of treated samples, plus the log2 of the mean of values of controls, all divided by 2. We also provided a PCA plot to show the distribution of LPS-treated and untreated samples. The PCA clearly shows two different cluster samples, one grouping LPS treated samples and one grouping controls. We made a PCA plot of normalized data, showing the first two principal components (PCs) of treated and untreated samples. Results were calculated using PCA tool R library (https://bioconductor.org/packages/release/bioc/vignettes/PCAtools/inst/doc/PCAtools.html#conduct-principal-component-analysis-pca) (Accessed on 30 September 2021).

### 4.3. Alignments and Phylogenetic Analyses

Multiple alignments of sequences were carried out using CLC (Version 7.0.0). Phylogenetic trees were designed in MEGA X maintaining the bootstrap value of 1000 bootstrap iterations (neighbour-joining method). The accession numbers are listed in Table 5.

### 4.4. qRT-PCR

The differential expression of *Cytochrome P450* LPS responsive genes was studied by qRT-PCR using the SYBR-Green method and the specific sets of primers listed in Table 6. qRT-PCR analysis was performed using an Applied Biosystems 7500 Real-time PCR system [34]. Differential expression was determined in a 25 μL PCR mixture containing 2 μL of cDNA converted from 250 ng of total RNA, 300 nM primer (forward and reverse), and 12.5 μL of Power SYBR-Green PCR MasterMix (Applied Biosystems, Waltham, MA, USA). Amplification specificity was tested by real-time PCR melting analysis. To obtain sample quantification, the 2−^ΔΔCt^ method was used, and the relative changes in gene expression were analyzed as described in the Applied Biosystems Use Bulletin N.2 (P/N 4303859). The transcript levels from different tissues were normalized to that of actin to compensate for variations in the amount of RNA input. Relative expression was determined by dividing the normalized value of the target gene in each tissue by the normalized value obtained from the untreated tissue. To examine the time course of the response, LPS-treated ascidians 4 replicates (*n* = 4) were examined at incremental post-inoculation time points (1, 2, 4, 8, 12, 24, and 48 h). Untreated ascidians (*naïve*) 4 replicates (*n* = 4) were used as controls.

Heatmap generation of real-time data was made using Heatmapper web tool (http://www.heatmapper.ca) (Accessed on 20 June 2021). It allows users to generate, cluster and visualize expression-based heat maps from transcriptomic data. To compute the heatmap, Complete linkage was applied as clustering algorithm, and Pearson correlation was used as the method of distance measurement. Additionally, a z-score is calculated. It is a measure that describes a value’s relationship to the mean of a group of values. Z-score is measured in terms of standard deviations from the mean [62].

### 4.5. Functional Enrichment Analysis

Gene enrichment of *C. robusta* transcripts produced by NGS was made through The Clusters of Orthologous Genes (COG) database (geneontology.org, release February 2021). The three Gene Ontology (GO) subcategories were investigated, and they are the following: (i) Biological Process (BP); (ii) Molecular Functions (MF); (iii) Cellular Components (CC); (iii) *p*-value and adjusted *p*-value thresholds were set to <0.05 to have statistically significant results.

The Protein Analysis Through Evolutionary Relationships (PANTHER GO-slim analysis tool) (pantherdb.org, 16.0 release) System connected to the COG database was used to expand the annotation data of the gene and protein families obtained from GO. The PANTHER “GO-slim” analysis mode was selected to have more reliable and accurate results compared to the GO “GO-complete” annotation mode.

### 4.6. miRNA–mRNA Target Interaction Prediction 

RNA–RNA interaction predictions (miRNA–mRNA target) were performed through the miRNA target interaction predictor (miRNATIP) algorithm [63]. The algorithm filters out wrongly predicted interactions, by means of the computation of the binding free energy (free energy threshold <−12Kcal/mol). This last step allows for the strengthening of the power of predicted interactions calculated by the algorithm. Indeed, as it is known, the lower the free energy of two paired RNA strands, the more energy is needed to disrupt this duplex formation, thus deducing that the stronger RNA–RNA binding is when the binding energy is low, and there is a more stable state thermodynamically. To test the best free energy thresholds in the miRNATIP algorithm we did some experiments in *C. elegans* and in *H. sapiens* to verify the most favorable ∆G values of binding sites, and they were <−6 and <−7kcal/mol. As some other scientific papers related to RNA–RNA interaction prediction tools used lower energy thresholds to evaluate RNA–RNA stability, we decided then to lower these energy thresholds to <−12kcal/mol energy values also in *C. robusta* samples [64].

### 4.7. Study of Evolution Pattern of C. robusta Homologues microRNA in Animal Genome

To study the evolution pattern of conserved *C. robusta* homologues microRNA in the animal genome, we used different web resources as MirGeneDB 2.0 (https://mirgenedb.org, MirGene DB 2.0 release) (Accessed on 21 July 2021); miREval 2.0 (http://mimirna.centenary.org.au/mireval/) (Accessed on 21 July 2021) and miRBase database (http://www.mirbase.org, release October 2018) (Accessed on 21 July 2021).

### 4.8. Network Construction and Visualisation 

To build interaction networks between DE genes linked to Cytochrome and inflammation and interacting miRNAs, different steps were achieved: first, both DE cytochrome and inflammation genes were downloaded in the STRING database (db) (string-db.org, release 11.5, accessed on 12 August 2021). STRING db, is an open-source platform for reconstructing interaction networks by using direct, indirect and functional interactions between molecules. In the second step, the two different networks previously produced (Cytochrome and inflammation) were imported into the Cytoscape app (https://cytoscape.org, release March 2017) (Accessed on 13 August 2021), to generate graph.ml files. 

Cytoscape is an open-source software platform for visualizing molecular interaction networks and biological pathways and integrating these networks with annotations. 

In the third step of the analysis, R package and R studio (https://www.r-project.org/, release October 2020) (Accessed on 17 August 2021); (https://rstudio.com/products/rstudio/, release October 2020) (Accessed on 17 August 2021) were used to integrate miRNA-targets networks from cytochrome and inflammation networks.

In particular, “igraph” package of R was used to analyses networks produced by STRING and to generate the image of integrated networks.

### 4.9. Transcription Factor Orthologue Identification and TF Binding Site Prediction

Cyp450 gene regulation by transcription factors was investigated. We searched for orthologue TFs in *C. robusta* through the use of different web services: National Center of Biotechnology Information (NCBI) (https://www.ncbi.nlm.nih.gov/gene, 244 release, accessed on 15 June 2021), orthologue database (orthoDB), (https://www.orthodb.org/v9, release v10.1) (Accessed on 20 August 2021), and REGULATOR web tool (https://www.bioinformatics.org/regulator, release 27.0) (Accessed on 20 August 2021).

Both orthoDB and REGULATOR collect and documents the transcription factors of metazoan species, between which *C. robusta*, giving information on the divergence in species evolution. The tools are also linked to other web resources such as InerPro, IntAct, Pfam, STRING, GO annotation, NCBI. 

Transcription Factor binding site prediction was also made to predict if orthologue TFs can potentially interact with genomic sequences of deregulated Cytochrome genes. The analysis was performed using the Site Tracking and Recognition (SiTaR) tool (https://sbi.hki-jena.de/sitar/, V 0.1) (Accessed on 24 August 2021). The method is based on the idea that it can be calculated several motifs with a given nucleotide content and a given number of mismatches in a random sequence. These predicted numbers can then be compared with the real occurrences of motifs in a query sequence. Moreover, this method uses a novel approach compared with position weight matrices (PWM) and Hidden Markow Models, and it is based on the research of motifs on input sequence, where for each motif found, scores are assigned depending on the non-randomness of the motif’s occurrence, the number of matching searching motifs and the number of mismatches. The method also has the advantage to significantly reduce the number of false-positive predictions significantly.

### 4.10. Statistical Methods

Fisher’s exact test performed statistical assessments of GO term enrichment and pathway analyses in combination with a robust false discovery rate (FDR) correction for multiple testing. The row *p*-value and FDR threshold were set as <0.05. 

Minitab 17 statistical software was used for the qRT-PCR data analysis. Statistical differences were estimated by one-way ANOVA, and the significance of differences among groups was determined by Tukey’s *t*-test. The level of significance was set at a *p*-value ≤0.05. The data are presented as the means ± SD (*n* = 4).

## Figures and Tables

**Table 1:** Deregulated Cytochrome families identified by NGS.

ENSEMBL ID	NAME	LogFC	LogCPM	*p*-Value	Adj *p*-Value	Log*p* Value	Chr. Position
ENSCING00000023704	cytochrome P450 2B10 (LOC101242210)	2.326276709	−0.954064903	7.85 × 10^−5^	0.001978725	4.104962459	Chromosome 11: 2,221,309–2,225,001 reverse strand.
ENSCING00000014704	cytochrome P450 2C15-like (LOC100186646)	2.08035981	0.149882558	5.87 × 10^−5^	0.001551545	4.23164033	Chromosome 10: 1,471,100–1,473,817
ENSCING00000017012	cytochrome P450 2J6-like (LOC100175185)	3.28873837	−1.420263798	2.46 × 10^−6^	0.000105594	5.608489757	Chromosome 8: 5,194,025–5,197,825 reverse strand.
ENSCING00000005903	cytochrome P450 2U1(LOC100185251)	1.511849854	0.613848759	0.002721531	0.035008689	2.565186709	Scaffold HT001236.1: 8,305–10,991
ENSCING00000004714	cytochrome P450 4B1-like (LOC100182965)	2.251530553	1.669810098	6.12 × 10^−6^	0.000231035	5.213430612	Chromosome 1: 7,364,051–7,366,204 forward strand
ENSCING00000006567	cytochrome P450 4F6-like (LOC100186171)	2.228936378	2.87892154	4.97 × 10^−6^	0.000194829	5.303654163	Chromosome 7: 1,548,748–1,552,535 forward strand.
ENSCING00000009298	cytochrome P450 2U1-like (LOC100182684)	-2.611064465	5.303935694	7.82 × 10^−8^	5.38 × 10^−6^	7.106747277	Chromosome 5: 2,876,599–2,880,776 forward strand.
ENSCING00000013919	cytochrome P450 2C42-like (LOC100184869)	-3.458407214	7.389835139	6.28 × 10^−12^	1.17 × 10^−9^	11.20175682	Chromosome 10: 3,837,453–3,843,016 reverse strand.

**Table 2:** Conserved Cytochrome P450 motifs in the *Ciona robusta*. “Length” indicates the total length of the translated protein sequence.

Cyp P450	Lengh	I-Helix [AG]G-x-[DE]T[TS]	K-Helix E-x-x-R	Meander Coil FDDER	Heme Loop F-x-x-G-x-R-x-C-x-G
2B10-like	365	AGTETS	ESLR		-------------CL-
2C15	509	AGTETS	KQLL	FRPER	FSVGLRSCIG
2C42	506	AGVETT	DQLX	FNPHR	FSIGPRYCMG
2U1	501	AGTDTT	EQLF	FKPDR	FNVGQRSCLG
2U1-like	506	AGTETT	LQLL	FKPER	FSVGPRQCLG
2J6	504	AGNETT	LQLC	FDPSR	FSLGPRQCIG
4B1	439	-	-DIE	FNPDR	FSAGSRNCIG
4F6	538	EGHDTT	KEIR	YDPER	FSAGPRNCIG

**Table 3:** Table shows miRNA-mRNA target interactions for deregulated genes linked to cytochrome (blue) and inflammation (red). All interactions showed in the table are divided for conserved and species-specific miRNAs. They are filtered for energy values and are ordered by number of interacting gene for each miRNA. Just results of miRNA-target interactions with more than two interacting targets are showed.

Conserved miRNAs	Target Gene ID				
cin-miR-196-3p	ENSCING00000004714	ENSCING00000009298	ENSCING00000005269	ENSCING00000013600	
	(*cyp450 4B1-like*)	(*cyp450 2U1-like*)	(*Il-17-3*)	(*Tgf-ß-na2*)	
cin-miR-92e-5p	ENSCING00000004714	ENSCING00000022988	ENSCING00000023704		
	(*cyp450 4B1-like*)	(*cyp450 2U1-like*)	(*cyp450 2C13, male-specific-like*)		
cin-let-7f-5p	ENSCING00000014704	ENSCING00000005269 (*Il-17-3*)			
	(*cyp450 4B1-like*)				
cin-let-7d-5p	ENSCING00000022988	ENSCING00000006967			
	(*cyp450 4F4-like*)	(*Il-17-1*)			
cin-let-7e	ENSCING00000004714 (*cyp450 4B1-like*)	ENSCING00000006967 (*Il-17-1*)			
cin-let-7d-3p	ENSCING00000013919 (*cyp450 2C42-like*)	ENSCING00000013600 (*Tgf-ß-na2*)			
cin-miR-200-3p	ENSCING00000008093	ENSCING00000023704			
	(*Tgf-ß*-*na1*)	(*cyp450 2C13, male-specific-like*)			
**Specie-Specific miRNAs**		**Target Gene ID**			
cin-miR-5596b-3p	ENSCING00000005903		ENSCING00000013919	ENSCING00000014704	ENSCING00000017012
	(*cyp450 2U1*)		(*cyp450 2C42-like*)	(*cyp450 4B1-like*)	(*cyp450 2J6-like*)
cin-miR-4085-3p	ENSCING00000022988		ENSCING00000014704	ENSCING00000006967	
	(*cyp450 4F4-like*)		(*cyp450 4B1-like*)	(*Il-17-1*)	
cin-miR-4036-3p	ENSCING00000009298		ENSCING00000013919	ENSCING00000008093	
	(*cyp450 2U1-like*)		(*cyp450 2C42-like*)	(*Tgf-ß*-*na1*)	
cin-miR-4200-3p	ENSCING00000006567		ENSCING00000017012		
	(*cyp450 4F6-like)*		(*cyp450 2J6-like*)		
cin-miR-4148-5p	ENSCING00000005903		ENSCING00000006567		
	(*cyp450 2U1*)		(*cyp450 4F6-like*)		
cin-miR-4116-5p	ENSCING00000005903		ENSCING00000006967		
	(*cyp450 2U1)*		(*Il-17-1*)		
cin-miR-4119-3p	ENSCING00000006567		ENSCING00000006967		
	(*cyp450 4F6-like)*		(*Il-17-1*)		
cin-miR-4011a-5p	ENSCING00000006567		ENSCING00000008093		
	(*cyp450 4F6-like)*		(*Tgf-ß*-*na1*)		
cin-miR-4064-5p	ENSCING00000013919		ENSCING00000008093		
	(*cyp450 2C42-like*)		(*Tgf-ß*-*na1*)		
cin-miR-5604-3p	ENSCING00000017012		ENSCING00000008093		
	(*cyp450 2J6-like*)		(*Tgf-ß*-*na1*)		
cin-miR-4020b-5p	ENSCING00000005269		ENSCING00000013600		
	(*Il-17-3*)		(*Tgf-ß-na2*)		
cin-miR-4065-3p	ENSCING00000006567		ENSCING00000013600		
	(*cyp450 4F6-like)*		(*Tgf-ß-na2*)		
cin-miR-4121-3p	ENSCING00000014704		ENSCING00000013600		
	(*cyp450 4B1-like*)		(*Tgf-ß-na2*)		
cin-miR-4203-3p	ENSCING00000022988		ENSCING00000013919		
	(*cyp450 4F4-like*)		(*cyp450 2C42-like*)		
cin-miR-4083-5p	ENSCING00000005903		ENSCING00000014704		
	(*cyp450 2U1)*		(*cyp450 4B1-like*)		
cin-miR-5596a-3p	ENSCING00000005903		ENSCING00000014704		
	(*cyp450 2U1)*		(*cyp450 4B1-like*)		
cin-miR-4001b-5p	ENSCING00000014704		ENSCING00000017012		
	(*cyp450 4B1-like*)		(*cyp450 2J6-like)*		
cin-miR-5605-3p	ENSCING00000005903		ENSCING00000017012		
	(*cyp450 2U1*)		(*cyp450 2J6-like)*		

**Table 4:** Table shows all miRNAs that are common to deregulated cytochrome genes and cytokines, filtered for low energy values.

Conserved miRNAS	Species-Specific miRNAs
cin-let-7d	cin-miR-3575-3p
cin-let-7e	cin-miR-4001b-1-3p
cin-let-7f-5p	cin-miR-4009a-3p
cin-miR-183-5p	cin-miR-4009b-5p
cin-miR-196-3p	cin-miR-4011a-5p
cin-miR-200-5p	cin-miR-4020b-3p
cin-miR-672	cin-miR-4036-3p
cin-miR-7-5p	cin-miR-4037-5p
cin-miR-92c-5p	cin-miR-4043-5p
cin-miR-92e-5p	cin-miR-4047-3p
	cin-miR-4058-5p
	cin-miR-4064-5p
	cin-miR-4064-5p
	cin-miR-4065-3p
	cin-miR-4077d-5p
	cin-miR-4102-5p
	cin-miR-4115-5p
	cin-miR-4116-5p
	cin-miR-4117-5p
	cin-miR-4119-3p
	cin-miR-4121-3p
	cin-miR-4148-5p
	cin-miR-4187-5p
	cin-miR-4207-3p
	cin-miR-4219-5p
	cin-miR-5596a-3p
	cin-miR-5596b-3p
	cin-miR-5603-3p
	cin-miR-5607-3p
	cin-miR-5609-5p
	cin-miR-5612-5p
	cin-miR-4219-5p

**Table 5:** Access numbers.

Name	GenBank no.
*Ciona robusta* Cyp P450 2B10-like	XP_026692382.1
*Branchiostoma floridae* Cyp P450 2J6-like	XP_035667220
*Branchiostoma floridae* Cyp 450 2U1-like	XP_035667221.1
*Homo sapiens* Cyp 450 2B6	NP_000758.1
*Homo sapiens* Cyp 450 1A1	NP_000490.1
*Homo sapiens* Cyp 450 2D6	NP_000097.3
*Homo sapiens* Cyp 450 26A1	NP_000774.2
*Homo sapiens* Cyp 450 4A22	NP_001010969.2
*Homo sapiens* Cyp 450 4A11	NP_000769.2
*Homo sapiens* Cyp 450 2C18	NP_000763.1
*Homo sapiens* Cyp 450 2A7	NP_000755.2
*Ciona robusta* Cyp 450 2C15-like	XP_002123518.1
*Branchiostoma floridae* Cyp 450 2C15-like	XP_035694363.1
*Drosophila melanogaster* C15	NP_476873.2
*Ciona robusta* Cyp 450 2C42-like	XP_018669209.2
*Sus scrofa* Cyp 450 2C42	NP_001161307.1
*Aplysia californica* Cyp 450 2C42	XP_012936173.2
*Crassostrea gigas* Cyp 450 2C42	XP_011452086.2
*Bos Taurus* Cyp 450 2C42	XP_005225699.1
*Ciona robusta* Cyp 450 2J6-like	XP_002129285.1
*Mus musculus* Cyp 450 2J6	NP_034138.3
*Haplochromis burtoni* Cyp 450 2J6	XP_014186874.1
*Xenopus tropicalis* Cyp 450 2J6	XP_002935636.1
*Strongylocentrotus purpuratus* Cyp 450 2J6	XP_001178133.3
*Ciona robusta* Cyp 450 2U1	XP_002119562.1
*Homo sapiens* Cyp 450 2U1	NP_898898.1
*Mus musculus* Cyp 450 2U1	NP_082092.2
*Ciona robusta* Cyp 450 2U1-licke	XP_026690452.1
*Homo sapiens* Cyp 450 4F2	sp|P78329.1|
*Homo sapiens* Cyp 450 4F3	sp|Q08477.2|
*Homo sapiens* Cyp 450 4B1	NP_001093242.1
*Mus musculus* Cyp 450 4B1	NP_031849.1
*Bos taurus* Cyp 450 4B1	NP_001069670.1
*Haplochromis burtoni* Cyp 450 4B1	XP_014190098.1
*Xenopus tropicalis* Cyp 450 4B1	XP_012817051.1
*Branchiostoma floridae* Cyp 450 4B1-like	XP_035680110.1
*Drosophila melanogaster* Cyp 450	AAC47424.1
*Ciona robusta* 4B1	XP_002125043.1
*Ciona robusta* 4F6	XP_002123011.3

**Table 6:** Primers used for qRT-PCR.

Gene	Primer Sequence (5′-3′)
*Mif1*	5′-GCTTGCAGCGCTTTTGATG-3′ 5′-AAACGGGTTCCAGAAACTCCTAA-3′
*Mif2*	5′-CCATGAAGCAACGAGGGAAA-3′ 5′-TTCTTGGCTGCGAGTTGGT-3′
*Cyp 2B10*	5′-CAAGGCCCAGCGTTTCAG-3′ 5′-CATTGCTGTGGGCTTCGAT-3′
*Cyp 2C15*	5′-CAACGACAAGCATCGAACTCA-3′ 5′-TTGGCGATAACAGGCATACCA-3′
*Cyp 2C42*	5′-TCGTCATTTTAGGTCGGTGATG-3′ 5′-TCAGTCATAGCTCGATACGAATACG-3′
*Cyp 2J6*	5′-TCCTAAATGGCAAGATCGCATA-3′ 5′-AAACTCCGTTCTCACCGATATTG-3′
*Cyp 2U1*	5′-TGGTCGAAGATCCGAACGA-3′ 5′-ACAACTGCTCTTTGTTCCACCAT-3′
*Cyp 2U1-like*	5′-AATGCAAAAATGGAGCAGAAAGT-3′ 5′-CCCGGCTCCCCATACG-3′
*Cyp 4B1*	5′-AAAACGAGCCCAACGTACCA-3′ 5′-TAAAGGTCCAAACCATGTTGTCA-3′
*Cyp 4F6*	5′-GGAGATGGTCTGTTGACAAGCA-3′ 5′-CAGGCGTTAGAAGCCTTCTGTT-3′
*Tgfβ*	5′-TTTCAGGGACCCAAAAACGA-3′ 5′-GCCAGCTATAATGACATCCAAGGT-3′
*Tnfα*	5′-GCCTCCCATAGACCGTTGTTAA-3′ 5′-CGGGACACCTTCAGCACAT-3′
*Il17-1*	5′- GCCGGGAACGTGACAGAA- 3′ 5′-GGCATGTTGATTGCGACCTT- 3′
*Il17-2*	5′-GTGTAGCGGGTGCATTGCT-3′ 5′-GGCACCGACTTCCCAACA-3
*Il17-3*	5′-CAAAGCGGAGCCTTCAATGT-3′ 5′-GCTTCTTTGCTCGACACTTGTG-3′
*Nf-kB*	5′-GCCGACGTACTGCTTTGCA-3 5′-GCCAGCCACCACGATGTT-3″
*Actin*	5′-TGATGTTGCCGCACTCGTA-3 5′-TCGACAATGGATCCGGT-3″

## Data Availability

Not applicable.

## Supplementary Materials

The following are available online at https://www.mdpi.com/article/10.3390/ijms222011141/s1.
